# Supplementary material for: Learning curve and surgical outcome of robotic assisted colorectal surgery with ERAS program
Source: Sci Rep. 2022 Nov 29;12:20566. doi: 10.1038/s41598-022-24665-w (PMC9709162; doi:10.1038/s41598-022-24665-w)
Supplement: Supplementary file 2 — Supplementary Information 2. [file 41598_2022_24665_MOESM2_ESM.zip › RAL Raw data-Ñ[▒K/049/OP N_ñw╝╨░O▒Kñσ.pdf]

W95 / W95

臺中榮民總醫院
手術室護理記錄

[redacted]

基本資料

- 室別： 開刀房-17室，手術科別： CRS，手術類別： 常規，日期： 2020/12/31
- 進入時間： 0725 ； 麻醉開始時間： 0730 ； Time Out時間： 0910 ； 劃刀時間： 0911 ； 手術結束時間： 1310 送出時間： 1330 ；
- 麻醉方式： GE
  診斷： Malignant tumor of sigmoid colon(PROVED, TATTO+)
  手術名稱： Robot Assisted Colectomy(L)
  手術局部用藥： nil
  術前預防性抗生素：
  - 抗生素名稱一： Veterin 2 gm IVD由麻醉人員給予
  - 時間： 0850
  - 上次簽章: 何宛臻

  ，追加預防性抗生素：
- 手術醫師： 蔣鋒帆，陳映菁，江世瑋
- 麻醉醫師： VS 陳永銘
-
  - 刷手護士A ： 李郁萱，時間： 0725，刷手護士B ： 鍾佩方，時間： 0725，接替刷手一： 廖子晴A，時間： 1200，接替刷手二： 李郁萱B，時間： 1230
  - 流動護士A ： 何宛臻，時間： 0725，接替流動一： 羅媗鈴，時間： 1200

皮膚狀況

A-V Shunt： 無
術前： 完整
記錄者： 上次簽章: 何宛臻
術後： 完整
記錄者： 羅媗鈴

手術準備

手術臥式 單一手術臥式

-
  - 手術臥式： 截石臥式
  - 使用附件： 溫毯 38 ℃，頭圈，其它： 頭架
  - 皮膚準備： Hibiscrub，2% Chlorhexidine
  - 電燒： 使用電燒: 電極片位置： 右大腿，記錄者： 上次簽章: 何宛臻

止血帶T： 無

植入物

- 植入物名稱/號碼/數量/部位： Coviden GIA 80-3.8mm x2釘.
  Ethicon stapler circular CDH29A x1釘/S-colon

，記錄者： 羅媗鈴

標本送檢

- 細菌培養 無
- 細胞學 無
- 冰凍切片 無
- 病理標本
  組織名稱一： S-Colon
  ，
  組織名稱二： distal cut end
  ，
  組織名稱三： IMA root
  ，更多組織名稱
  -

- ○ 組織名稱四： jejunum
- 截肢 無

標本交付 標本： 4 袋 , 標本： 0 盒 , 標本： 0 瓶 , 細胞學： 0 管 , Culture Tube： 0 管 , 被交付醫師： CR 陳映菁 ,

- 記錄者： 羅媗鈴
- 時間： 1315

傷口術後敷料

傷口敷料：　　傷口敷料： 無

- 請於圖上標示部位：

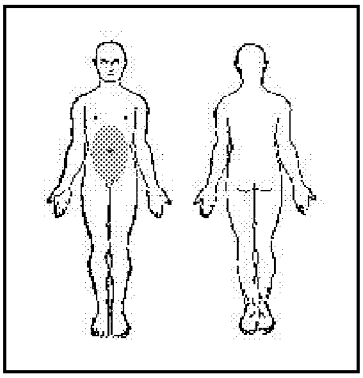

- 傷口敷料： mepore

導管

導管	天數	導管資訊	插入日期	插入時間	更換日期	拔除日期	拔除時間	拔除原因	預計拔除/更換	預計拔除/更換日期
Foley(1)	1	號碼:16 Fr.; 部位:膀胱;	2020/12/31	0830						
Silicone NG Tube(1)	1	號碼:16 Fr.; 深度:50cm; 部位:胃;	2020/12/31	0850						
Double Lumen CVC(1)	1	號碼:8 Fr; 深度:15cm; 部位:jugular (右); 象限:RU;	2020/12/31	0825						
A-line (1)	1	號碼:20 G; 部位:左手;	2020/12/31	0810						

用物計數

- 手術中出血量： minimal c.c.
- 手術中尿量： 1400 c.c.
-
  - 其它： 無

用物計數： 單一切口

- 1.器械： 正確 , 2.縫針： 正確 , 3.刀片： 正確 , 4.紗布： 正確 , 5.腹部墊： 未使用 , 6.紗球： 未使用 , 7.PEANUTS ： 未使用 , 8.紗條： 未使用 , 9.絲帶： 未使用 , 10.針頭： 未使用 , 11.Vessel loop： 未使用 , 12.Boots： 未使用 , 13.Jelco： 未使用 , 14.棉條： 未使用 ,
  - 第一次：
    - 刷手護士 ： 廖子晴
    - 流動護士： 羅媗鈴
    - 時間： 1245

,
  - 第二次：
    - 刷手護士 ： 廖子晴
    - 流動護士： 羅媗鈴
    - 時間： 1255

,
  - 第三次：
    - 刷手護士 ： 廖子晴
    - 流動護士： 羅媗鈴
    - 時間： 1305

備註

備註： OLYMPUS 腹腔鏡NO.6

- 記錄者： 羅媗鈴 上次簽章: 何宛臻
- 時間： 1200

填寫護理人員 羅媗鈴

正式摘要

	W95 / W95

doctorRank:B
